# Potassium extrusion by plant cells: evolution from an emergency valve to a driver of long‐distance transport

**DOI:** 10.1111/nph.20207

**Published:** 2024-10-27

**Authors:** Dorsaf Hmidi, Florence Muraya, Cécile Fizames, Anne‐Aliénor Véry, M. Rob G. Roelfsema

**Affiliations:** ^1^ Institut des Sciences des Plantes de Montpellier, Univ Montpellier, CNRS, INRAE, Institut Agro, Campus SupAgro‐INRAE 34060 Montpellier Cedex 2 France; ^2^ Molecular Plant Physiology and Biophysics, Julius‐von‐Sachs Institute for Biosciences, Biocenter Würzburg University Julius‐von‐Sachs‐Platz 2 D‐97082 Würzburg Germany

**Keywords:** action potential, *Characeae* internode cell, guard cell, ion extrusion, phloem loading, potassium, stomata, xylem loading

## Abstract

The ability to accumulate nutrients is a hallmark for living creatures and plants evolved highly effective nutrient transport systems, especially for the uptake of potassium (K^+^). However, plants also developed mechanisms that enable the rapid extrusion of K^+^ in combination with anions. The combined release of K^+^ and anions is probably an ancient extrusion system, as it is found in the *Characeae* that are closely related to land plants. We postulate that the ion extrusion mechanisms have developed as an emergency valve, which enabled plant cells to rapidly reduce their turgor, and prevent them from bursting. Later in evolution, seed plants adapted this system for various responses, such as the closure of stomata, long‐distance stress waves, dropping of leaves by pulvini, and loading of xylem vessels. We discuss the molecular nature of the transport proteins that are involved in ion extrusion‐based functions of plants and describe the functions that they obtained during evolution.

**Table:** 

	Contents	
	Summary	69
I.	Introduction	69
II.	A safety valve uncovered in the *Characeae*	70
III.	Guard cells	73
IV.	Long‐distance signals in land plants	76
V.	Long‐distance nutrient transport in xylem and phloem	78
VI.	Outlook	80
	Acknowledgements	83
	References	83

## I. Introduction

It is plausible that life has emerged in an environment with high K^+^ concentrations, possibly in the K^+^‐rich sheets of clay particles of the mineral mica (Hansma, 2022). This would explain why the biochemistry of life is adapted to high levels of K^+^ inside the cells (Danchin & Nikel, 2019). Later in evolution, organisms will have moved into diverse environments such as oceans, in which the K^+^ concentration is only 10 mM, while that of Na^+^ is *c*. 470 mM. This represents a challenge for the cells, since the chemical interaction of Na^+^ with biomolecules tends to have a negative impact on their structure, unlike the interaction with K^+^, despite the similar chemical nature of both monovalent cations (Benito *et al*., 2014). As a result, the activity of many enzymes requires high concentrations of K^+^, but low levels of Na^+^ (Cui & Tcherkez, 2021). During evolution, marine organisms learned to maintain high cytosolic K^+^ concentrations, in the order of 100 mM, in an environment that is poor in K^+^, but rich in Na^+^. It thus was essential for these organisms to be able to accumulate K^+^ in their cytosol, while extruding Na^+^.

In comparison with marine plants, land plants often need to cope with even lower extracellular K^+^ levels and can even survive in soils with less than 10 μM free K^+^ (Römheld & Kirkby, 2010; Zörb *et al*., 2014). At such low K^+^ levels (concentration gradient > 10^4^), K^+^ will be a limiting factor for growth and agricultural soils that are intensively used therefore need to be resupplied with K^+^‐containing fertilizers (Zörb *et al*., 2014). Plants have evolved two types of K^+^ uptake systems, in order to thrive on soils with a wide variety of K^+^ levels. At high K^+^ levels in the soil, plants will mainly use K^+^‐channels of the voltage‐dependent cNBD‐containing K^+^ channels (previously known as ‘Shaker‐type K^+^ channels’) such as AKT1 (see Box 1 for abbreviations), while they switch to KUP/KT/HAK transporters that enable K^+^‐uptake in symport with H^+^, in soils with low K^+^ levels (Véry *et al*., 2014; Jegla *et al*., 2018).

Box 1 **List of abbreviations**

ALMT, aluminum‐activated malate transporters; APs, action potentials; *At*PP2CA, *Arabidopsis thaliana* protein phosphatases type 2C with homology to ABI1/ABI2; BLINK 1, blue Light‐induced K^+^ channel 1; CRISPR‐Cas9, clustered regularly interspaced short palindromic repeats associated protein (Cas)9; DiBAC4, Bis‐(1,3‐dibutylbarbituric acid) trimethine oxonol; *E*_K_, equilibrium potential for K^+^; ER, endoplasmic reticulum; GCaMP6, cpEGFP‐CalModulin‐M13‐Peptide 6; GECIs, genetically encoded Ca^2+^ indicators; GORK, gated outwardly rectifying K^+^ channel; *Gt*ACR1, *Guillardia theta* light‐gated anion channelrhodopsin 1; KCR2, Kalium channelrhodopsin 2; Kup/HAK/KT, K^+^ uptake permease/high‐affinity K^+^ transporter/K^+^ transporter; MAMP, microbe‐associated molecular pattern; MbDio, marine bacterial β‐carotene 15,15′‐dioxygenase; *MAXI‐K/BK* channel gene, calcium‐dependent potassium BK (Maxi‐K) channels; NPF, nitrate transporter/peptide transporter family; NRT1, nitrate transporter 1; OST1, Open Stomata 1; pHuji, pH‐sensitive red fluorescent protein; PP2Cs, protein phosphatases type 2C; PTORK, *P. tremula* outward rectifying K^+^ channel; PYR/PYR‐like (PYL), pyrabactin resistance‐like proteins; QUAC1, quick anion channel 1; RCARs, regulatory components of ABA receptor; R‐GECO1, red fluorescent genetically encoded Ca^2+^ indicator 1; R‐type, rapid anion channel; SKOR, Stelar K^+^ outward rectifier; SLAC1, slow anion channel 1; SLAH3, slow anion channel homolog 3; SnRK2.6, Snf1‐related protein kinase 2.6; SUT1, sucrose transporter 1; TPK, two‐pore K^+^ channel; VACs, vessel associated cells; VFK1, *Vicia faba* K^+^ channel 1; *Vv*K3.1, *Vitis vinifera* Shaker K^+^ channel 3.1.

Plant cells thus will accumulate K^+^ and other osmotically active solutes at a much higher concentration than in the extracellular medium. As a result, an osmotically driven flux of water into the cells will provoke a turgor pressure, which exerts a force via the plasma membrane on the cell wall. Due to the turgor pressure, the plant cells remain stiff and drive cell expansion when the cell wall extends. However, a high turgor pressure can also impose a threat to a cell, as a sudden increase in the pressure exerted on the cell wall can cause cell rupture. Such a rapid rise in turgor of cells will occur in nature, during a heavy rainfall when the nutrients in the soil are being diluted, or in coastal regions where seawater gets mixed with fresh water.

It is likely, that the first plants exposed to rapid decreases in osmolarity of their environment have evolved the ability to rapidly release ions. This capability enabled them to avoid cell rupture and thus represented a major advantage for survival in an environment where the osmolarity can rapidly drop. Later in evolution, vascular plants used these mechanisms to drive solute transport trough their vascular systems and control movements in pulvini of leaves and stomata.

Below, we will describe the combined K^+^ and anion extrusion mechanism, first discovered in algae, which further evolved in vascular plants, to support specialized functions.

## II. A safety valve uncovered in the *Characeae*

### 1. Adaptations to changes in osmotic conditions of *Lamprothamnium* spp.

Algae that are living at seashores are well capable to cope with large and rapid changes in the osmotic conditions of their environment. On sunny days, the osmolarity of salt lakes will increase above that of seawater, while it can also rapidly drop during a heavy rainfall. The ability to adapt to such large changes in osmolarity was intensively studied with *Lamprothamnium* spp. (Kishimoto & Tazawa, 1965; Bisson & Kirst, 1980a,b). It turned out that *Lamprothamnium papulosum* can maintain a stable turgor pressure, even when the osmolality of its growth medium ranged from 150 to 1350 mOsm (Bisson & Kirst, 1980b). In the cells of *L. papulosum*, the altered osmotic conditions were mainly compensated through the uptake and release of K^+^ and Cl^−^.

The *Lamprothamnium* species belong to the *Characeae*, of which most species are found in freshwater. The *Characeae* are famous for their giant internodal cells that can get several centimeters long and have been a favorite object for electrophysiology in early days (Osterhout & Hill, 1930; Umrath, 1930). In *Lamprothamnium*, changes in osmotic conditions were shown to have a strong impact on the membrane potential of internodal cells (Reid *et al*., 1984). Stimulation with hyperosmotic conditions, caused a depolarization of the plasma membrane for *c*. 10 min., followed by a hyperpolarization to potentials that were more negative than before stimulation. A hypoosmotic shock also provoked a depolarization, but in this case, the cells remained depolarized for periods over 1 h (Reid *et al*., 1984).

It is likely that the hypoosmotic‐induced prolonged depolarization correlates to rapid release of K^+^, as explained in further detail below. This mechanism enables *Lamprothamnium* cells to extrude K^+^ and Cl^−^ in response to a drop in osmolarity, which will occur during heavy rainfall. Such a response can be regarded as a ‘safety valve’ that prevents cell rupture. The safety valve system probably existed already in early plants, even before the *Characeae* separated from the lineage that gave rise to land plants.

### 2. Action potentials in Characeae

**Fig. 1:**
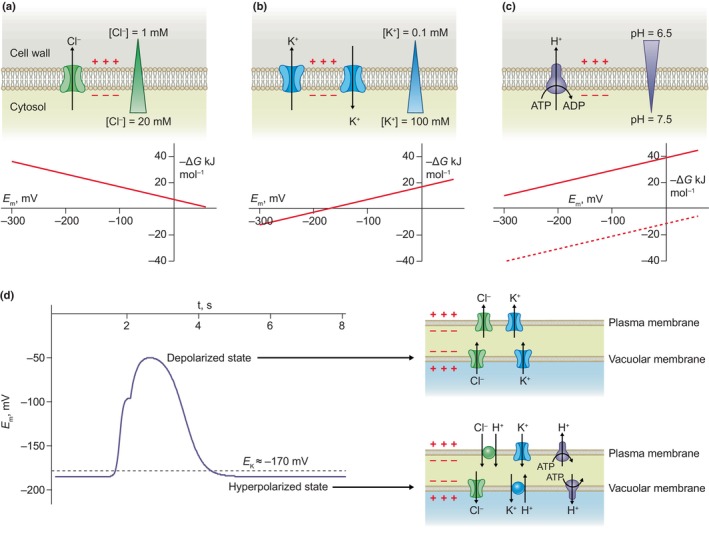
Relation between ion fluxes and action potentials in internode cells of the Characeae. (a–c) *Upper panel*s, schematic representation of the ion transport, membrane potential, and ion gradient across the plasma membrane of *Chara* internode cells, (a) shown for chloride (Cl^−^), (b) potassium (K^+^), and (c) H^+^ transported by the H^+^‐ATPase. Note that the efflux of Cl^−^ is facilitated by anion channels, the efflux, and uptake of K^+^ by K^+^ channels and the extrusion of H^+^ by H^+^‐ATPases. *Lower panels*, the negative change in Gibbs energy (−Δ*G*) plotted against the membrane potential, calculated for the ion transport proteins depicted in the upper panels. Note that negative values of ΔG indicate an efflux, while positive values support uptake of the respective ions. H^+^ fluxes are shown uncoupled of ATP hydrolysis (dotted line) and coupled with ATP hydrolysis (continuous line) at a 1 : 1 stochiometry. Note that coupling with ATP hydrolysis shifts the line of H^+^ upward (see Nicholls & Stuart, 2002). (d), Action potential of internode cells (graph on the left side, redrawn according to Beilby & Coster, 1979), during which the direction of Cl^−^ and K^+^ fluxes, temporarily reverses, as indicated for K^+^ by the striped line that represents the calculated K^+^ Nernst potential. During the depolarization, K^+^ and Cl^−^ are released by ion channels in the plasma and vacuolar membranes (upper cartoon on the right side). Upon membrane hyperpolarization, K^+^ is taken up via K^+^ channels (lower cartoon on the right side), while uptake of Cl^−^ across the plasma membrane requires co‐transport with H^+^. Sequestration of K^+^ into the vacuole depends on antiport with H^+^, while the vacuolar membrane potential supports uptake of Cl^−^ via anion channels into the vacuole. Both the plasma membrane and vacuolar membrane possess H^+^‐ATPases that link hydrolyzation of ATP to H^+^ transport.

Freshwater *Characeae* (related to *Lamprothamnium*) have been a favorite subject for studies on so‐called ‘action potentials’ (APs; Fig. 1), which were first recorded by Osterhout & Hill (1930) in *Nitella flexis* internodal cells. Even though the plant AP is *c*. 1000 times slower than in animals, APs in both kingdoms are characterized by uniform transient changes of the membrane potential (Kisnieriene *et al*., 2022). They represent an ‘all or nothing’ response that occurs, once a certain stimulus threshold has been reached. In the *Characeae*, action potentials can be evoked by various triggers, such as electrical and mechanical stimuli, or light pulses. Moreover, action potentials are elicited upon an increase in the turgor pressure of internodal cells, which can be imposed with a pressure probe (Zimmermann & Beckers, 1978; Beilby, 2007). The AP thus may be related to the ‘safety valve’ responses described above for *Lamprothamnium* spp., which enables aquatic cells to adapt to rapid changes in osmotic conditions.

Studies in the 1970s revealed that internodal cells can either be in the depolarized‐, or hyperpolarized state, with respect to the electrical potential across the plasma membrane (Spanswick, 1972; Richards & Hope, 1974). In the depolarized state, the plasma membrane potential values are close to the so‐called ‘Equilibrium potential for K^+^’ (*E*_K_) (Box 2), and as a result, the membrane potential strongly depends on the extracellular K^+^ concentration (Fig. 1). By contrast, cells have membrane potential more negative than *E*_K_ in the hyperpolarized state and here the activity of the H^+^‐ATPase plays a dominant role (Spanswick, 1972). As explained in Fig. 1, the difference between membrane potential and the *E*_K_ value will determine whether a cell takes up K^+^ via K^+^ channels, or uses channels to release K^+^ into the extracellular medium.

Box 2 **Ion transport, Gibbs energy, and Nernst potential**

The transport of ions through pores in a membrane (as occurs in ion channels) depends on the difference in ion concentration and the electrical charge difference across the membrane (membrane potential, see also Fig. 1). Consequently, the Gibbs energy change that determines the direction of ion flow can be calculated with an equation that has two components (Nicholls & Stuart, 2002): one dependent on the concentration difference of the ion (K^+^ in Eqn 1) and an electrical component, which depends on the membrane potential.

(Eqn 1) $$\Delta G=\underset{\text{concentration component}}{\underbrace{2.3R\times T\times \log_{10}\left[\frac{{\left[K^{+}\right]}_{\text{out}}}{{\left[K^{+}\right]}_{\mathrm{cyt}}}\right]-z_{K^{+}}}}\times \underset{\text{electrical comp}.}{\underbrace{F\times E_{M}}}$$

Eqn 1, dependence of changes in Gibbs energy (ΔG) on the K^+^ concentration difference, and membrane potential across a membrane. *R* is the gas constant (8.31), *T* is the absolute temperature (293°K = 20°C), ${\left[K^{+}\right]}_{\text{out}}$ is the external K^+^ concentration, ${\left[K^{+}\right]}_{\mathrm{cyt}}$ is the cytosolic K^+^ concentration, *z*_K+_ is valence of K^+^ (+1), *F* is the Faraday constant (9.65 × 10^4^), and *E*_m_ is the membrane potential (mV).

If the net current of K^+^ across the membrane is null, no change in Gibbs energy (ΔG) will occur, and Eqn 1 can be converted to Eqn 2.

(Eqn 2) $$E_{K}=2.3\;\frac{\;R\times T\;}{z_{K^{+}}\times F}\times \log_{10}\left[\frac{{\left[K^{+}\right]}_{\text{out}}}{{\left[K^{+}\right]}_{\mathrm{cyt}}}\right]$$

Eqn 2, Equilibrium potential of K^+^ (*E*_K_). All parameters are the same as in Eqn 1.

Eqn 2 is known as the K^+^ equilibrium potential, or Nernst potential of K^+^. Note that this potential corresponds to the voltage that is recorded with an ion‐selective electrode (like the pH electrode). These electrodes are connected to high‐resistance amplifiers, of which the electric current is negligible. Consequently, the measured potential equals the equilibrium potential of the ion that is conducted by the ion‐selective ‘membrane’ in the electrode.

In a review that summarizes 70 yr of work on internodal cells, Beilby (2007) concludes that during the AP, the membrane polarization transiently switches from the hyperpolarized‐ to the depolarized state. This response is induced by the release of Ca^2+^ from internal stores, which causes a rise in the cytosolic Ca^2+^ concentration, and in turn activates plasma membrane anion channels that depolarize the plasma membrane (Box 2; Fig. 1). Because of the transient change in membrane potential, the cells will temporarily release Cl^−^ and K^+^ (Beilby, 2007). In contrast to the action potentials in animal cells, the plant response does not involve Na^+^ currents, which makes sense, as land plants are often growing at conditions with very low levels of Na^+^.

The molecular nature of the channels that enable the release of K^+^ and Cl^−^ from *Chara* cells has not yet been resolved. However, the *Chara braunii* genome only encodes one channel with homology to the Arabidopsis voltage‐dependent cNBD K^+^ channels SKOR and GORK (Box 3; Supporting Information Fig. S1; Table S1; Gaymard *et al*., 1998; Ache *et al*., 2000; Hosy *et al*., 2003; Jegla *et al*., 2018; Nishiyama *et al*., 2018), which suggests that this channel enables K^+^ extrusion across the plasma membrane. Likewise, *C. braunii* only possesses a single Aluminum‐Activated Transporters (ALMT) channel gene (Box 3; Fig. S2; Table S1; Nishiyama *et al*., 2018) that may encode the Ca^2+^‐activated anion channel, which depolarizes *Chara* cells during the action potential. The difficulty to transform *Chara* cells has so far hampered experiments that tested the proposed roles of both channels. However, the CRISPR‐Cas techniques may offer new possibilities to modify gene expression in the giant internode cells and resolve some outstanding questions related to nature and role of plant action potentials that have fascinated scientists for almost 100 yr.

Box 3 **Evolutionary relationship of ion channels involved in K^+^ extrusion**

**K^+^ channels**

Two types of K^+^‐efflux channels have been identified in Arabidopsis, named SKOR and GORK, which belong to the voltage‐gated cNBD K^+^ channels (previously also named ‘plant Shaker‐like’ channels; Jegla *et al*., 2018). For simplicity, these channels will be named SKOR/GORK channels, even though they have received other names in some other species. The SKOR/GOKR channels which alter their voltage‐dependent gating properties, in response to altered extracellular K^+^ concentrations (Gaymard *et al*., 1998; Ache *et al*., 2000; Hosy *et al*., 2003; Véry *et al*., 2014; Drain *et al*., 2020). In the freshwater alga *Klebsormidium nitens*, seven homologs of the SKOR/GORK genes are present. In all other plants, the gene family is reduced to one or two members, while they are absent in the moss *Physcomitrium patens* (Table 1).

Slowpoke/MAXI‐K/Big‐K/K_Ca_ (BK) channels are also voltage‐regulated K^+^ channels (Sancho & Kyle, 2021), but in contrast to the GORK/SKOR channels, their open probability is not affected by the extracellular K^+^ concentration. These channels were identified in the vacuolar membrane of *Chara* cells (Luhring, 1986; Tyerman & Findlay, 1989; Homblé & Véry, 1992), while transient expression of a *M. polymorpha* BK channel in *Nicotiana benthamiana* resulted plasma membrane localization (Sussmilch *et al*., 2021), which suggests that BK channels could be active in both membranes. However, the BK channels are absent in flowering plants (Table 1).

TPK/KCO channels are voltage‐independent K^+^‐selective channels, which mainly occur in the vacuole of Arabidopsis cells (Maathuis, 2011; Dabravolski & Isayenkov, 2021). An exception in Arabidopsis is TPK4, which was located to the pollen tube plasma membrane (Becker *et al*., 2004). Whereas most plant species have several TPK genes, only one gene can be found in the genomes of *M. polymorpha*, *C. braunii*, and *K. nitens* (Table 1). TPK1‐channels thus seem to represent an evolutionary conserved gene family that predominantly controls the release of K^+^ from vacuoles and most likely adopted cell‐specific functions in mosses, lycophytes, ferns, and seed plants.

**Anion channels**

The number of SLAC/SLAH genes seems to be relatively stable in all clades of the green lineage (Dreyer *et al*., 2012), with the exception of *C. braunii* (Table 1), in which these channels were lost. The absence of SLAC/SLAH genes can be explained by the lack of changes in the osmotic conditions that this freshwater species will encounter. In such stable environmental conditions, a rapid release of osmolytes may not be needed and these channels were omitted.

Whereas SLAC/SLAH channels are likely involved in mass extrusion of inorganic anions, ALMT channels can also transport small organic anion, such as malate and citrate, both in the plasma membrane and vacuolar membrane (Dreyer *et al*., 2012; Sharma *et al*., 2016). Only a single ALMT gene is found in *Chara braunii*, which suggests that it encodes an R‐type channel that could be essential for generation of action potentials. Most other nonvascular plants have several ALMT channels, while a clear enlargement of this gene family occurs in vascular plants and 18 genes are found in *Picea abies*. It is likely that the expansion of the ALMT genes enables the vascular plants to control the transport of specific organic anions.

ALMT‐type channels also are involved in the release of anions from the vacuole and these genes cluster in Arabidopsis in a clade with ALMT3‐6 and ALMT9 (Dreyer *et al*., 2012; Sharma *et al*., 2016). Homologous genes are found in other vascular plants, but not in nonvascular plants. Future studies thus may reveal if the ALMTs of nonvascular plants are localized in the plasma membrane, vacuolar membrane, or both membrane systems.

**Table 1:** Number of genes encoding K^+^, or anion channels of a specific type, listed for several model species.

Clade	Species	SKOR/ GORK	BK Channels	TPK/KCO	SLAC/ SLAH	ALMT
Charophyte algae	*Klebsormidium nitens*	7	2	1	4	2
Characeae	*Chara braunii*	1	1	1	0	1
Liverworts	*Marchantia polymorpha*	1	3	2	2	4
Mosses	*Physcomitrium patens*	0	3	4	4	5
Spikemosses	*Selaginella moellendorffii*	2	4	5	5	2
Gymnosperms	*Picea abies*	1	1	3	4	18
Basal magnoliophyta	*Amborella trichopoda*	2	0	3	4	7
Dicots	*Arabidopsis thaliana*	2	0	6	5	14
Monocots	*Oryza sativa*	2	0	4	9	10

### 3. Role of vacuole in internodal cells

Action potentials in internodal cells are not restricted to the plasma membrane, but also concern a transient change in vacuolar membrane potential (Findlay & Hope, 1964). A coordinated response of both membranes is logical, since the vacuole represents the largest compartment in internode cells, and during prolonged ion efflux, as occurs in the *Lamprothamnium* spp. (as described in the previous section), most of the ions released by the cell will originate from the vacuole. Ion fluxes across the plasma membrane thus need to be synchronized with those across the vacuolar membrane. It is likely that the transient elevation of cytosolic Ca^2+^ level during the action potential (Kikuyama & Shimmen, 1997) serves in communication between both membranes. During the rise of the cytosolic free Ca^2+^ concentration, vacuolar anion channels are activated and the vacuolar membrane transiently hyperpolarizes from *c*. −20 to −40 mV (Shimmen & Nishikawa, 1988; Kikuyama & Shimmen, 1997; Berecki *et al*., 1999).

The vacuolar membrane of internodal cells harbors at least two K^+^ channel types (Luhring, 1986; Tyerman & Findlay, 1989; Homblé & Véry, 1992), of which the large conductance K^+^ channel is activated by high cytosolic Ca^2+^ concentrations (Laver & Walker, 1991). It is likely that this channel is encoded by the *MAXI‐K/BK* channel gene that was identified in the *C. braunii* genome (Box 3; Fig. S3; Table S1; Nishiyama *et al*., 2018). The second K^+^ channel could be a TPK channel, of which homologs in Arabidopsis serve as Ca^2+^‐activated K^+^ selective channels in vacuoles (Box 3; Fig. S4; Table S1; Gobert *et al*., 2007; Latz *et al*., 2007). During the action potentials, thus both anion‐ and K^+^ channels activate and will enable the simultaneous release of K^+^ and Cl^−^ into the cytosol (Fig. 1). The conductance of anion channels is likely to exceed that of the K^+^ channels, since the vacuolar membrane hyperpolarizes and thus probably shifts to the Nernst potential of Cl^−^ (Laver & Walker, 1991), based on an *c*. six times higher Cl^−^ concentration in the vacuole, compared with the cytosol (Tazawa *et al*., 1974).

One of the functions of the APs thus is to coordinate the K^+^‐release across the vacuolar‐ and plasma membrane. In addition, APs will also synchronize other physiological processes in the large internode cells, such as cytoplasmic streaming and photosynthesis (Bulychev *et al*., 2023).

## III. Guard cells

### 1. Osmotically driven movements of stomata

Guard cells control the aperture of stomatal pores that are located in the leaf surface and enable the uptake of CO_2_ for photosynthesis. These pores also allow the evaporation of water and therefore stomata close during drought, to prevent wilting. Stomatal movements are osmotically driven, through changes in the ion content of the guard cells (Raschke *et al*., 1988). If guard cells accumulate osmolytes, water will be drawn into their interior and cause cell swelling. As a result, the guard cell size will increase, which causes it to bend and force the opening of the pore in between two neighboring guard cells, while the reverse process leads to stomatal closure (Roelfsema & Hedrich, 2005). Stomatal closure thus depends on the extrusion of osmolytes and thus may have the same evolutionary origin, as the safety valve of *Lamprothamnium*.

**Fig. 2:**
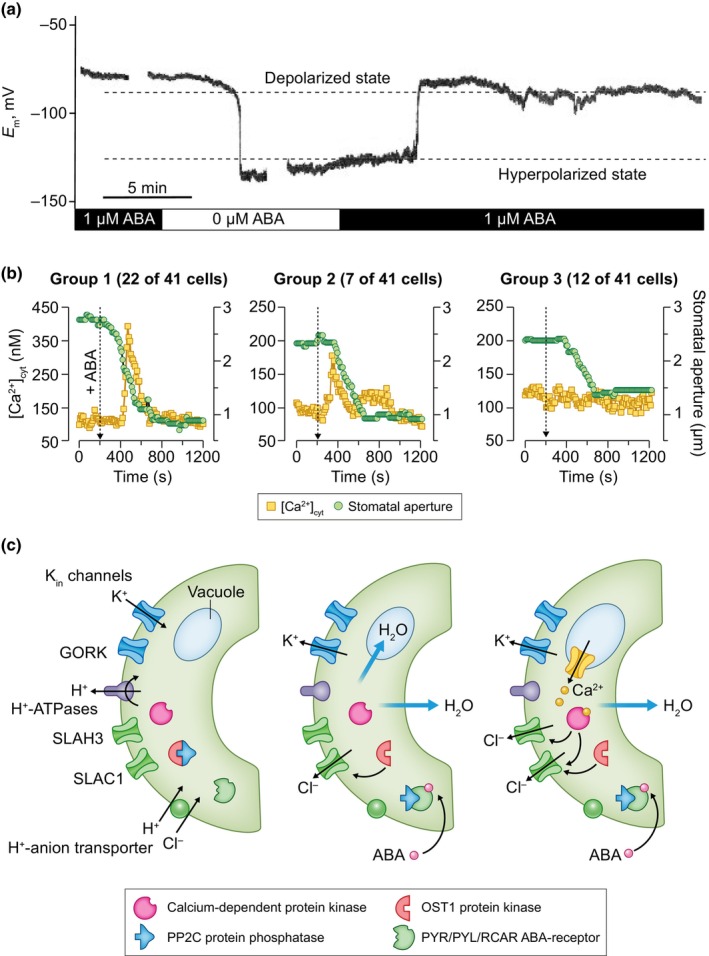
The drought hormone abscisic acid (ABA) provokes K^+^‐extrusion in guard cells. (a) Membrane potential trace of a *Vicia faba* guard cell in an intact leaf, exposed to a solution containing 1 μM ABA at starting conditions, followed by the temporal removal of ABA, as indicated by the bar below the graph. Note that removal of ABA evoked a reversible shift from the depolarized, to the hyperpolarized state. Unpublished data from Victor Levchenko, University of Würzburg, Germany. (b) Simultaneous recordings of the cytosolic Ca^2+^ concentration in single Arabidopsis guard cell and the aperture of the stoma in which this guard cell was present. The stomata were stimulated with ABA after 200 s, as indicated by the arrow. Note that 54% of cells displayed a rise in the cytosolic Ca^2+^ concentration during stomatal closure (Group 1), whereas ABA provoked a Ca^2+^ signal before closure of the pore in 17% of experiments (Group 2) and the remaining pores closed in the absence of a cytosolic Ca^2+^ signals (Group 3; 29% of cells). Data from Huang *et al*. (2019). (c). Schematic representation of the sequence of events that explains the response of guard cells in Group 1 (according to (b)). Left drawing, in the absence of ABA the H^+^‐ATPase generates a membrane potential and an H^+^ gradient that enables the uptake of K^+^ via K_in_ channels and Cl^−^ by an H^+^‐anion transporter. Middle drawing ABA binds to the PYR/PYL/RCAR ABA receptors that inhibit PP2C protein phosphatases, the protein kinase OST1 is released from inhibition and can activate Slow Anion Channel 1 (SLAC1). Because of activation of SLAC1, the guard cell shifts from the hyperpolarized to the depolarized state (as in (a)) and K^+^ is extruded into the cell wall via the GORK channel. Due to the loss of osmolytes from the cytosol, the guard cells lose water, and the vacuole initially swells. Right drawing, in response to swelling, the vacuole will release Ca^2+^ and provoke a cytosolic Ca^2+^ signal that activates both SLAC1 and SLAH3.

Just as in internode cells, guard cells can occur in two membrane potential states (Thiel *et al*., 1992; Roelfsema & Prins, 1998). In the hyperpolarized state, the membrane potential is more negative than the Nernst potential for K^+^, and drives the uptake of K^+^, via K^+^‐selective uptake channels such as KAT1, KAT2, and AKT1 (Anderson *et al*., 1992; Sentenac *et al*., 1992; Szyroki *et al*., 2001; Lebaudy *et al*., 2008). The extrusion of K^+^, via the Arabidopsis GORK efflux channel (Ache *et al*., 2000; Hosy *et al*., 2003), occurs in the depolarized state, in which the membrane potential is slightly positive of the K^+^ Nernst potential (Thiel *et al*., 1992; Roelfsema & Prins, 1998). Guard cells can switch from the hyperpolarized‐ to the depolarized state in a manner that resembles that of the AP in internode cells of the *Characeae* (Thiel *et al*., 1992; Blatt & Armstrong, 1993; Roelfsema & Prins, 1998; Fig. 2). However, in intact plants, guard cells normally remain in the de‐ or hyperpolarized state for longer periods of time (Roelfsema *et al*., 2001, 2002). The responses of guard cells are thus more similar to the long‐lasting membrane potential changes that are provoked by hypoosmotic conditions in *Lamprothamnium*, than the rapid APs that were extensively studied in *Chara* and in *Nitella* spp.

Guard cells thus utilize the hyperpolarized state to provoke stomatal opening and switch to the depolarized state to close stomata (Fig. 2). Below, we will discuss the properties of the ion transporters that are active in either of both states and examine their evolutionary origin.

### 2. Hyperpolarized state

The hyperpolarized state is also referred to as ‘pump state’, as it depends on a high activity of the plasma membrane H^+^‐ATPase, also named ‘proton pump’. This protein uses the energy released by the conversion of ATP, to ADP and phosphate, to translocate a single H^+^ from the cytosol to extracellular space (Fig. 1c; Briskin & Hanson, 1992; Buch‐Pedersen *et al*., 2009). Because of the low stoichiometry (1 H^+^/1 ATP), the plasma membrane proton pump can generate a large proton motive force and drive the membrane potential to values as negative as −240 mV, provided that inward K^+^ channels are blocked (Lohse & Hedrich, 1992; Buch‐Pedersen *et al*., 2009). However, at physiological conditions, the outward current of the proton pump is compensated by an inward current through K^+^ channels and limits the degree of hyperpolarization (Roelfsema & Prins, 1998; Roelfsema *et al*., 2001). In other words, the proton pump drives the uptake of K^+^ via ion channels, by generating a hyperpolarized cell membrane.

It is likely that the protons extruded into the guard cell wall are taken up again, via co‐transport with anions. In the case of NO_3_^−^, NRT1.1 was shown to facilitate this transport in Arabidopsis (Guo *et al*., 2003), while a similar transporter has been postulated for Cl^−^ but was not studied in further detail (Jezek & Blatt, 2017). The NRT1.1 transporter was reported to facilitate inward currents (Tsay *et al*., 1993), which suggests that at least two protons are taken up with each NO_3_^−^ ion, although, theoretically the K^+^ and anion uptake into guard cells could be more efficient, if the plasma membrane anion transporters would have a stoichiometry of 1H^+^ per anion. With the use of 1 ATP, guard cells would extrude 1H^+^, allowing the uptake of 1K^+^‐ion driven by the membrane potential and 1 anion (NO_3_^−^, or Cl^−^) using the H^+^‐concentration gradient.

In addition to the K^+^‐uptake channels, guard cells also express H^+^–K^+^ co‐transporters of the KUP/HAK/KT‐type (Maser *et al*., 2002). These transporters were reported to affect stomatal movements (Osakabe *et al*., 2013), but their role in ion transport in guard cells has not been resolved. Potentially, these H^+^–K^+^ co‐transporters could facilitate K^+^ uptake into guard cells, at low extracellular K^+^ levels.

### 3. Depolarized state

Most plant cells expand irreversibly to support plant growth and only have a limited ability to shrink. Guard cells are an exception to this rule, as they reversibly accumulate and extrude ions, to support stomatal opening and closure. Experiments with intact *Vicia faba* plants, in which single guard cells were studied with microelectrodes, revealed that stomatal closure is initiated by a transition from the hyperpolarized‐ to the depolarized state (Roelfsema *et al*., 2001). These transitions coincided with the activation of slow‐type (S‐type) anion channels in the plasma membrane of guard cells, in response to darkness (Roelfsema *et al*., 2001). A similar stomatal closure response could be induced with high atmospheric CO_2_ levels (Roelfsema *et al*., 2002) and the drought stress hormone abscisic acid (ABA) (Fig. 2; Roelfsema *et al*., 2004). Based on these experiments, it was concluded that stomatal closure is initiated through the activation of S‐type anion channels, which leads to the extrusion of anions (NO_3_^−^, or Cl^−^), as well as a depolarization of the plasma membrane, and provokes the extrusion of K^+^ via GORK‐type channels (Ache *et al*., 2000; Hosy *et al*., 2003; Nguyen *et al*., 2017; Drain *et al*., 2020). As a result, guard cells in the depolarized state will extrude both anions and K^+^, lose turgor, and cause stomatal closure (Fig. 2).

The important role of S‐type anion channels for stomatal movement was supported by studies with Arabidopsis mutants that lack the Slow Anion Channel 1 (SLAC1) (Negi *et al*., 2008; Vahisalu *et al*., 2008). These plants have wide open stomata, which do not close in response to ozone, high atmospheric CO_2_ and low air humidity (Merilo *et al*., 2013). Guard cells also express the SLAC1‐homologous channel SLAH3 (Demir *et al*., 2013), and a loss of both SLAC1 and SLAH3 results in stomata that lack S‐type anion channels and fail to close in response to ABA and the microbe‐associated molecular pattern (MAMP) flg22 (Guzel Deger *et al*., 2015).

SLAC‐type channels probably evolved early in evolution, as these channels share homology to dicarboxylate transporters expressed in bacteria and archaea and are present in the genome of *K. nitens* (Box 3; Fig. S5; Table S1; Dreyer *et al*., 2012). However, SLAC‐type channels are not found in the genome of *Chara braunii*, which suggest that they are not involved in the action potentials found in these cells. Instead, an ALMT channel could be crucial for these membrane potential changes, as discussed above.

In addition to the S‐type channels, rapid (R‐type) anion channels have also been discovered in guard cells (Keller *et al*., 1989). It was postulated that R‐type channels are encoded by ALMT genes, based on the finding that loss of the *ALMT12*/*QUAC1* gene caused a lower activity of R‐type channels and slowed down stomatal closure in darkness (Meyer *et al*., 2010). However, a further loss of guard cell ALMT genes could not eliminate the R‐type anion conductance (Jaslan *et al*., 2023), which suggests that so far unidentified gene products must contribute to this anion channel conductance. ALMT channels thus seem to have an important role in dark‐induced stomatal closure, which may be related to the ability of these channels to transport organic anions (Sharma *et al*., 2016). However, further research is required to elucidate the specific roles of AMLT‐ and SLAC/SLAH‐type channels.

Experiments with the light‐activated anion channel *Gt*ACR1 showed that opening of anion channels in the plasma membrane of guard cells is sufficient to provoke stomatal closure (S. G. Huang *et al*., 2021; Zhou *et al*., 2021). *Gt*ACR1 belongs to the family of channelrhodopsins, which encode ion channels that are activated with blue or green light pulses. If *Gt*ACR1 is expressed in guard cells of tobacco, activation with blue light depolarizes the guard cell plasma membrane and leads to the extrusion of anions and K^+^ and closure of stomata (S. G. Huang *et al*., 2021). This study thus showed that the activation of anion channels in the plasma membrane of guard cells is sufficient to close stomata. Due to the depolarization of the plasma membrane, GORK channel will automatically activate and enable the extrusion of K^+^ (S. G. Huang *et al*., 2021).

### 4. Signaling pathways controlling stomatal movements

Various environmental signals, such as light, the atmospheric CO_2_ concentration, air humidity, and MAMPs, affect stomatal movements (Kollist *et al*., 2014; Munemasa *et al*., 2015; Jezek & Blatt, 2017). Because of the number of signals that feed in on this response, it is likely that a complex network exists in guard cells, which controls stomatal movements. The discussion of all details of signal transduction pathways in guard cells that have been discovered is beyond the scope of this paper. Instead, we will limit our discussion on two major pathways that are important for stomatal closure.

The protein kinase Open Stomata 1 (OST1), also known as SNF1‐related protein kinase 2.6 (SnRK 2.6), plays a central role in stomatal closure, as loss of its activity leads to stomata that are insensitive to ABA, CO_2_, ozone, and MAMP (Mustilli *et al*., 2002; Merilo *et al*., 2013; Guzel Deger *et al*., 2015). OST1 can directly interact with, and activate, the SLAC1 anion channel and thereby provoke ion extrusion and stomatal closure (Geiger *et al*., 2009; Lee *et al*., 2009). At conditions that lead to stomatal opening, OST1 is inhibited by several 2C‐type protein phosphatases (PP2Cs) and SLAC1 remains inactive (Fig. 2). However, in the presence of ABA these PP2Cs from a complex with a class of proteins named pyrobactin (PYR)/ PYR‐like (PYL) proteins, or regulatory components of ABA receptor (RCARs) (Ma *et al*., 2009; Park *et al*., 2009; Cutler *et al*., 2010). Due to the formation of this complex, the PP2Cs are downregulated and OST1 gets active, leading to stimulation of SLAC1. The activation of OST1 also depends on a group of Raf‐like kinases (Lin *et al*., 2020; Takahashi *et al*., 2020), which are likely to regulate OST1 in response to osmotic signals (Saruhashi *et al*., 2015).

The OST1‐dependent signaling mechanism described above is independent of Ca^2+^ signals, which are encoded by temporal elevation of the cytosolic Ca^2+^ level in guard cells. However, such Ca^2+^ signals can activate slow anion channels (Schroeder & Hagiwara, 1989; Geiger *et al*., 2010, 2011) and have been linked to ABA‐induced stomatal closure (McAinsh *et al*., 1990; Gilroy *et al*., 1991; Allen *et al*., 2000). Huang *et al*. (2019) studied Arabidopsis guard cells and found that ABA induced a transient rise in the cytosolic Ca^2+^ concentration in approximately two out of three stomata that are stimulated with ABA (Fig. 2b). It was concluded that ABA can close stomata through a mechanism that is independent of Ca^2+^ signals (probably based on the OST1‐SLAC1 interaction, as described above), but Ca^2+^ signals enhanced the efficiency by which the stress hormone closed stomata (Fig. 2c; Huang *et al*., 2019).

Guard cell Ca^2+^ signals are likely to address at least two targets that can speed up stomatal closure. First of all, they can activate the SLAH3 anion channel in the plasma membrane, which is not targeted by OST1 (Fig. 2c; Geiger *et al*., 2011). In addition, Ca^2+^ signals may activate two‐pore K^+^ (TPK) channels in the vacuolar membrane of guard cells (Gobert *et al*., 2007; Latz *et al*., 2007). The activation of TPK1 is likely to depolarize the vacuolar membrane of guard cells and provoke a release of K^+^ via TPK1 (Gobert *et al*., 2007). As a result of the depolarization, the vacuolar membrane can enable the release of Cl^−^ via ALMT9 (De Angeli *et al*., 2013), which was shown to be important for stomatal movements. The cytosolic Ca^2+^ signals thus may synchronize events at the plasma membrane and the vacuolar membrane (Cubero‐Font & De Angeli, 2021), and thereby speed up stomatal closure.

Whereas the TPK channels (in the vacuolar membrane) and SKOR/GORK channels (in the plasma membrane) thus seem to represent the key elements to control the release of K^+^ in flowering plants, BK (MAXI‐K) channels were also found in the vacuolar membrane of *Chara*, where they may have a similar function. These channels are predestinated to mediate massive K^+^ efflux upon membrane depolarization, owing to their large conductance (Box 3; Fig. S3; Table S1; Luhring, 1986; Tyerman & Findlay, 1989; Homblé & Véry, 1992).

## IV. Long‐distance signals in land plants

### 1. Common nature of APs in plants

APs do not only occur in the *Characeae*, but also have been found in other branches of the evolutionary tree. In liverworts, true APs, with an all or nothing response, seem to be widespread (Kisnieriene *et al*., 2022), but only few seed plants seem to be able to transmit APs. In the famous species *Mimosa pudica* and *Dionaea muscipula* (the Venus flytrap), APs provoke rapid movements, like folding of leaflets in *M. pudica* and closure of the insect traps of *D. muscipula*. Experiments with ion‐selective electrodes and ion channel inhibitors showed that the start of the AP in the liverwort *Conocephalum conicum* is marked by an increase in the cytosolic Ca^2+^ level. It is very likely that the elevated Ca^2+^ level leads to the activation of plasma membrane anion channels, just as in *Chara* internode cells (Trebacz *et al*., 1994, 1997).

### 2. Ca^2+^ signals and movements in excitable seed plants

The Ca^2+^ signals which are triggered during APs could recently be monitored with genetically encoded Ca^2+^ indicators (GECIs) in *D. muscipula* and *M. pudica* (Suda *et al*., 2020; Hagihara *et al*., 2022). In *M. pudica*, the touch‐induced APs triggered strong increases in the cytosolic Ca^2+^ concentration of the pulvini, just before the leaflets started to drop. During this response, water is moving from the lower, to the upper part of the pulvinus (Tamiya *et al*., 1988). Apparently, the AP in *M. pudica* provokes a large Ca^2+^‐induced loss of turgor in cells at the lower side of the pulvinus. Such a response may be due to the activation of anion channels in cells of the lower part of the pulvinus. In this case, the short depolarization during the AP would be translated into a long‐lasting Ca^2+^ signal that triggers anion and K^+^ extrusion in pulvini cells. The activation of anion channels in pulvini of *M. pudica* awaits further study, but leaf movements in *Samanea saman*, strongly correlated with the expression of the anion channel *SsSLAH1* in the pulvini (Oikawa *et al*., 2018).

**Fig. 3:**
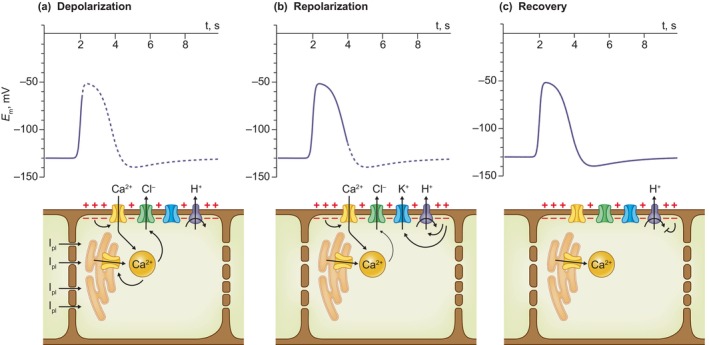
Action potential of the Venus flytrap (redrawn according to Scherzer *et al*., 2022, fig. 2f). Upper graphs, the propagated part of the action potential is displayed as a solid trace, while the remaining is shown as a dashed curve. The response is separated in a depolarization phase (a), followed by a repolarization phase (b), and a recovery phase (c). The action potential is probably provoked by (a), a depolarization of the neighboring cell, which provokes a current through plasmodesmata and depolarizes the plasma membrane of the flytrap cell that causes activation of Ca^2+^‐permeable channels. The initial rise of the cytosolic Ca^2+^ concentration is amplified by the endoplasmic reticulum and activates plasma membrane anion channels, thereby reinforcing the depolarization. (b) In response, voltage‐activated K^+^ channels and H^+^ ATPases are activated and cause a repolarization. (c) During the recovery, the activity of the H^+^ ATPases slowly decreases to the pre‐stimulus value.

In the Venus flytrap, the sequence of events that provokes trap closure seems to be slightly more complicated than in *M. pudica*. Insects that are walking on a lobe of a trap will bend one of the three trigger hairs and thereby will stimulate stretch‐activated ion channels in the podium of the hair. The stretch‐activated Cl^−^ channel (Flycatcher1 (FLYC1)) has been identified in triggers hairs (Procko *et al*., 2021), which will depolarize cells at the base of the trigger hairs. However, it is unclear how such a depolarization can cause Ca^2+^ signals, as depolarization‐activated Ca^2+^ channels have not been found in plants. Just as described for *M. pudica* above, a transient rise of the cytosolic Ca^2+^ is thought to be essential for the propagation of APs (Scherzer *et al*., 2022). Such Ca^2+^ signals will activate plasma membrane anion channels, which is most likely required for signal propagation (Fig. 3; Scherzer *et al*., 2022).

During the depolarization phase of the AP, the depolarization‐activated K^+^ channel *Dm*GORK will open and cause a repolarization of the plasma membrane toward the K^+^ equilibrium potential (Box 2; Fig. 3). Shortly thereafter the *Dm*GORK channels are expected to deactivate again and now the H^+^‐ATPase (*Dm*AHA4) can fully repolarize the plasma membrane potential (Fig. 3). During the action potential K^+^ will thus be extruded, but because of the short period of depolarization, it is unlikely that this causes a bulk flow of K^+^. Instead, the *Dm*GORK channels seem to have a different function, since they initiate the recovery phase of the action potential.

The Ca^2+^ signal during a single AP is not sufficient to provoke trap closure but if a second action potential is provoked within 10 s, the cytosolic Ca^2+^ concentration in the trap is rising further and exceeds a threshold value that causes trap closure (Suda *et al*., 2020). In this case, the trap closure is initiated by the loss of turgor of cells on the outer surface of the trap (Forterre *et al*., 2005). This causes a rapid change in the shape of the lobes from outward, to inward bending arcs. The movement of the flytrap resembles a ‘snap‐buckling’ mechanism, which explains how the trap can close in *c*. 0.1 s.

APs in *D. muscipula* not only trigger a fast closure of the trap but also further responses that ensure the digestion of the prey. Normally, a captured insect will keep on moving and bending the trigger hairs in the closed trap, thereby provoking further APs (Böhm *et al*., 2016). The flytrap can count these signals and only starts to activate sodium uptake transporters after five sequential APs. Here, the APs seem to be no longer linked to K^+^ extrusion responses. Instead, the APs in this case provoke jasmonate‐dependent responses, in a similar way to ‘variation potentials’ that are initiated by herbivore attack in various seed plants, including Arabidopsis (Mousavi *et al*., 2013; Böhm *et al*., 2016; Hedrich & Roelfsema, 2023). It was recently shown that these variation potentials also occur in the liverwort *Marchantia polymorpha*, but in this species, no jasmonate signaling could be detected (Beraldo & Alboresi, 2024; Sanmartín *et al*., 2024; Watanabe *et al*., 2024). It thus seems as if the action potentials and variation potentials are transmitted via an evolutionary conserved mechanism, but the connection to jasmonate‐dependent defense responses has occurred more recently, possibly with the occurrence of vascular plants.

### 3. Genes encoding components of the apparatus that transmits APs

Despite of the large genetic distance between Chara, liverworts, and seed plants, the APs thus seem to be based on a common mechanism. This suggests that the critical molecular components are present in most plants, even though APs have not been detected in most seed plants. Most likely these plants have the genes required for excitability, but they do not express them in such a way that APs can be generated. This phenomenon could be nicely illustrated with *D. muscipula*, in which leaves and early stages of the trap are not excitable, and the trap only gets excitable in a late mature state (Scherzer *et al*., 2022).

## V. Long‐distance nutrient transport in xylem and phloem

### 1. SKOR mediates K^+^ extrusion into xylem vessels

Xylem vessels are built by cells that undergo programmed cell death, to give rise to xylem tubes, which form a continuum from the root to the shoot (Ruzicka *et al*., 2015). Within these tubes, a flow of water and solutes can either be driven by tension, due to transpiration, as well as a positive pressure build‐up in the root system (Schenk *et al*., 2021). In case of high air humidity, the transpiration rate of leaves will be negligible, and the xylem flow will solely dependent on the ‘positive root pressure’. The activity of this system is exemplified by ‘guttation’, a process in which plants exudate droplets via hydathodes at their leaf margins (Singh, 2016; Schenk *et al*., 2021).

Because mature xylem is devoid of protoplasts, the root pressure must originate from cells that are in contact with the xylem vessels. These xylem parenchyma cells, or vessel‐associated cells (VACs) are likely to extrude K^+^ into the xylem vessels via K^+^‐selective efflux channels (Wegner & Raschke, 1994; Roberts & Tester, 1995). In Arabidopsis, this channel is encoded by the Stelar K^+^ Outward Rectifier (*SKOR*) gene and mutants that have lost SKOR showed a 40% reduction in their K^+^ content (Gaymard *et al*., 1998). More recently, it was shown that the K^+^ efflux channels *Os*K5.2 and *Sl*SKOR have a similar function in rice and tomato, respectively (Nguyen *et al*., 2017; Zhou *et al*., 2022; Nieves‐Cordones *et al*., 2023).

The ability of SKOR to load the xylem may be dependent on a polar localization of this plasma membrane protein on the side that is adjacent to the xylem vessels. Such a localization was found for the SKOR homolog of poplar (PTORK) (Langer *et al*., 2004; Arend *et al*., 2005). It is thus likely that xylem parenchyma cells funnel K^+^ release into the xylem, with the use of the SKOR‐like K^+^ channels.

### 2. Role of anion channels in xylem loading

The release of K^+^ by SKOR‐like efflux channels requires a depolarization of the plasma membrane, to values positive of the K^+^ Nernst potential, just as described for GORK in guard cells, above. So far, direct measurements of the membrane potential of xylem parenchyma cells have not been reported, but instead the xylem potential (or trans‐root potential) was studied (Dunlop, 1982; de Boer *et al*., 1983; Wegner *et al*., 1999; Mian *et al*., 2011). The xylem potential represents the electrical potential difference between the xylem vessels and root medium and thus is the sum of the electrical potentials from the root epidermal‐ to the xylem parenchyma cells (de Boer, 1999). Based on xylem potential studies, it can be concluded that membrane potential of xylem parenchyma cells ranges from −50 to −150 mV (Dunlop, 1982; de Boer, 1999). In cells with a membrane potential of −50 mV, it is likely that anion channels are active, whereas the value of −150 mV is probably achieved in cells with low anion channel activity.

In patch clamp experiments with xylem parenchyma cells, the activity of S‐type anion channels was detected (Wegner & Raschke, 1994; Köhler & Raschke, 2000), and in Arabidopsis, these channels are probably encoded by *SLAH2* and *SLAH3* (Maierhofer *et al*., 2014; Cubero‐Font *et al*., 2016). Whereas the SLAH2 channel is selective for NO_3_^−^ (Maierhofer *et al*., 2014), SLAH3 can conduct both NO_3_^−^ and Cl^−^. This suggests that SLAH3 channels provide a general mechanism for anion extrusion, while SLAH2 enables plants to specifically load NO_3_^−^ into the xylem. Cells in contact with xylem also express the *SLAH1* gene, which does not form functional anion channels, but can convert SLAH3 in an active state (Cubero‐Font *et al*., 2016). It is thus likely that the simultaneous expression of *SLAH1* and *SLAH3* brings xylem parenchyma cells in a persistent depolarized state.

**Fig. 4:**
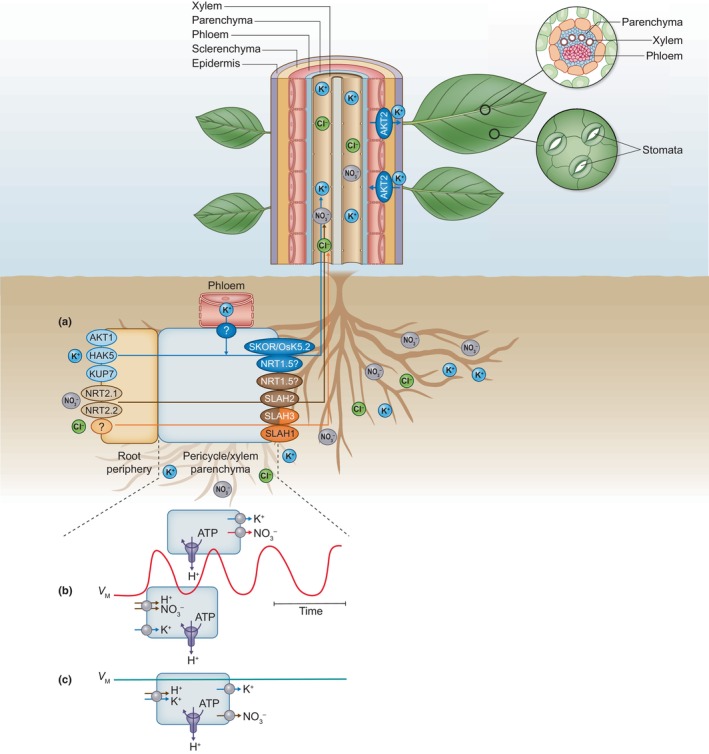
Secretion of K^+^ and anions to xylem vessels in Arabidopsis and rice. (a) K^+^ is taken up from the soil at the root periphery by AKT1 channels and KUP/KT/HAK transporters, while NO_3_^−^ is taken up by NRT1, or NRT2‐type transporters. The ions reach the pericycle and xylem parenchyma, from where they are extruded by ion‐selective efflux channels and transporters into the xylem sap. The release of K^+^ by SKOR channels (OsK5.2 in rice) requires a depolarized plasma membrane, which is imposed by the extrusion of NO_3_^−^ through the SLAH2 channel, and SLAH3, activated by SLAH1, which conducts both NO_3_^−^ and Cl^−^. Additionally, NRT1.5 may function in the secretion of NO_3_^−^, or K^+^, into the xylem. In phloem cells, the AKT2 channel contributes to the setting of the membrane potential and K^+^ loading in source tissues but the secretion mechanism out of phloem cells in sink tissues is not established. (b, c) Two hypotheses are proposed regarding the ion‐secretion mechanism: (b) The membrane of xylem parenchyma cells oscillates between a depolarized and a hyperpolarized state. Accumulation of K^+^ occurs during the hyperpolarized state while K^+^ release to the xylem vessels occurs during the depolarized state. (c) Alternatively, the continuous activity of SLAH1 and SLAH3 brings xylem parenchyma cells in a persistent depolarized state, while the activity of H^+^‐coupled K^+^ uptake systems enables the accumulation of K^+^ into the depolarized cells.

Because of the lack of membrane potential recordings on xylem parenchyma cells, it is not clear whether these cells tend to switch between a depolarized and hyperpolarized state (Fig. 4), as was found for internode and guard cells. A hyperpolarized state would be required, if the xylem parenchyma cells use K^+^‐uptake channels to accumulate K^+^ from the walls of adjacent cells. However, these cells may also use H^+^–K^+^ co‐transporters proteins to accumulate K^+^, such as the members of the KUP/KT/HAK family (Yang *et al*., 2014; Han *et al*., 2016), or obtain K^+^ via symplastic connections from neighboring cells. In the latter two models, the K^+^ supply to xylem parenchyma cells would be independent of the membrane potential and a continuous extrusion of K^+^ into xylem vessels would be possible at stable depolarized membrane potentials (Fig. 4).

### 3. Link between anion uptake and xylem loading

A strong connection between the transport of anions and K^+^ was also discovered in experiments with a member of the Nitrate transporter 1/Peptide transporter Family (NPF) (Léran *et al*., 2014). Loss of NPF7.3 (NRT1.5) reduced the concentrations of NO_3_^−^ and K^+^ in the shoot of seedlings, grown on medium with low K^+^ levels (Li *et al*., 2017) as well as the K^+^‐uptake of mature plants (Drechsler *et al*., 2015). The *NPF7.3* gene is highly expressed in root pericycle cells, which suggests that the encoded transporter has an important function in xylem loading (Lin *et al*., 2008).

Even though it is obvious that NPF7.3 has an impact on long‐distance transport of K^+^ and NO_3_^−^ (Lin *et al*., 2008; Drechsler *et al*., 2015; Li *et al*., 2017), its transport properties are still debated. Whereas Lin *et al*. (2008) conclude that NPF7.3 facilitates symport of H^+^ and NO_3_^−^, the data of Li *et al*. (2017) suggest antiport of H^+^ and K^+^. Based on these contrasting hypotheses, NPF7.3 may function in the uptake of NO_3_^−^ in pericycle (Lin *et al*., 2008), or enable the extrusion of K^+^ into the xylem (Li *et al*., 2017). In the latter scenario, K^+^ thus would be extruded by plant cells by a newly discovered mechanism, which does not depend on K^+^ extrusion channels like the SKOR/GORK and BK channels.

### 4. Phloem unloading

In most of the literature, phloem transport is linked to the release and uptake of carbohydrates into sieve tubes (Peters & Knoblauch, 2022). However, a recent study with maize plants that lack the SUT1 sucrose transporter, revealed that the loss of sucrose uptake, leads to an increase in the K^+^ concentration in sieve tubes (Babst *et al*., 2022). This suggests that the solute flow, normally provoked by sucrose gradients, can also be maintained by uptake of K^+^ into sieve tubes. Please note that in contrast to xylem vessels, sieve tubes possess a plasma membrane and are connected via plasmodesmata to phloem companion cells. K^+^‐loading of the phloem thus occurs through uptake of K^+^ into these cells, in contrast to loading of the xylem vessels that is dependent on K^+^ efflux from xylem parenchyma cells (as described in the previous section).

It is likely that the AKT2 channel (also named AKT3 or AKT2/3) plays a role in both the loading and the release of K^+^ from the phloem (Fig. 4). The AKT2 channel gene is highly expressed in phloem cells of aerial plant tissues (Deeken *et al*., 2000; Lacombe *et al*., 2000) and encodes a pH‐sensitive K^+^ channel (Marten *et al*., 1999; Lacombe *et al*., 2000). In line with the supposed role in phloem unloading, AKT2 and the homologous channels VFK1 and *Vv*K3.1, of *Vicia faba* and grapevine, respectively, are particularly expressed in aerial sink tissues (Deeken *et al*., 2000; Lacombe *et al*., 2000; Ache *et al*., 2001; Nieves‐Cordones *et al*., 2019).

In contrast to SKOR and GORK, AKT2 is not activated by depolarization of cell membrane, but instead can either occur in a voltage‐independent ‘mode 2’, or ‘mode 1’, in which it is activated by hyperpolarization (Chérel *et al*., 2002; Michard *et al*., 2005; Dreyer *et al*., 2017). The dephosphorylation of AKT2 by the AtPP2CA protein phosphatase causes a transition to mode 2 and the predicted role of AKT2 in the extrusion of K^+^ in sink tissues would take place in this voltage‐independent mode. The current of K^+^ from phloem cells, has to be accompanied by an ion current in the opposite direction (for instance by efflux of anions) but so far, it is not known how this is accomplished.

Phosphorylation of AKT2 will convert the channel to mode 1, where it opens only at membrane hyperpolarization that drives K^+^ uptake (Chérel *et al*., 2002; Michard *et al*., 2005; Dreyer *et al*., 2017; Y. N. Huang *et al*., 2021). This regulatory mechanism thus may enable phloem cell to disable K^+^ efflux via AKT2. Alternatively, the transition between both states was suggested to provide a mechanism that maintains sugar transport at condition of temporal ATP shortness causing a low activity of H^+^‐ATPases (Gajdanowicz *et al*., 2011; Dreyer *et al*., 2017).

## VI. Outlook

### 1. Evolution of K^+^‐extrusion mechanisms

The papers discussed in this review clearly indicate that an extrusion system for K^+^ has existed in a common ancestor of green plants, which has been inherited by modern species. In Arabidopsis, the SLAC/SLAH channels appear to serve as the major anion release channels that also depolarize the plasma membrane, thereby driving the K^+^ extrusion via SKOR/GORK channels. In line with this conserved ion extrusion system, SKOR/GORK channels are found in all model species, just as the SLAC/SLAH channels with the exception of *Chara braunii* (Box 3) This not only implicates that APs in *C. braunii* depend on another type of anion channel (most likely an ALMT channel) but also that *C. braunii* may be unable to rapidly adjust its osmotic value through the bulk release of K^+^ and anions.

Future experiments should clarify whether the *Chara* ALMT channel is indeed required for the initiation of APs and if SLAC/SLAH channels are present in the related *Lamprothamnium* species that are capable of rapidly releasing large quantities of K^+^ and Cl^−^ (Bisson & Kirst, 1980b). So far, such studies were hampered by the lack of genome data and difficulties to transform these algae (Kurtovic *et al*., 2024).

The role of cytosolic Ca^2+^ signals in the coordination of ion fluxes may also be answered by the repression of genes that encode components of this signaling module. For instance, a loss of the single TPK1 gene in *Chara* may prevent the vacuole of replenishing the K^+^ and Cl^−^ ions that are extruded by internode cells during the AP. An even stronger impact would be expected in *Lamprothamnium* spp. in which the extrusion of K^+^ and Cl^−^ can continue longer than 1 h (Reid *et al*., 1984). In guard cells, a similar Ca^2+^‐dependent coordination system for ion fluxes at the plasma‐ and vacuolar membranes has been postulated. However, stomatal closure, and the associated K^+^‐extrusion, can be evoked via the Ca^2+^‐independent OST1 protein kinase. Future experiments may reveal, whether OST1 also addresses the TPK1 channels at the vacuole, or if these channels are only activated during responses in which Ca^2+^ signals are triggered. It is feasible that the large changes in osmotic value of guard cells, which occur during stomatal closure, may trigger Ca^2+^ release from the vacuole (Voss *et al*., 2016), which in turn can activate TPK channels in the vacuolar membrane (Gobert *et al*., 2007; Latz *et al*., 2007).

### 2. Fluorescent reporters

Xylem and phloem strands are normally deeply imbedded in root or shoot tissues, which complicates studies on their K^+^‐extrusion mechanisms. The use of genetically encoded sensors that are expressed in specific cell types of these transport systems is likely to overcome the limitations that are encountered with classic single‐cell approaches. As K^+^‐efflux is often associated with Ca^2+^ signals, the specific expression of Ca^2+^ indicators like R‐GECO1 and GCaMP6 (Zhao *et al*., 2011; Chen *et al*., 2013; Waadt *et al*., 2017) will be a major step to study xylem and phloem cells in action in their natural environment. In addition to the well‐known GECIs, various other protein‐based sensors have been developed, which can be used to detect various solutes in intact cells, such as cytosolic [H^+^], [K^+^], and [NO_3_^−^] (Sadoine *et al*., 2023).

Based on the availability of fluorescent proteins that emit light over the whole spectrum of visible light (Rodriguez *et al*., 2017), even multiple sensors can be used in combination in a single experiment (Waadt *et al*., 2021). This enables the interrelation of several intracellular factors such as the cytosolic Ca^2+^ concentration and pH (Li *et al*., 2021), the cytosolic pH and anion level (Demes *et al*., 2020), or other combinations (Waadt *et al*., 2020). Moreover, the sensors can be targeted to specific intracellular compartments, which allows the simultaneous recording of the cytosolic and endoplasmic reticulum Ca^2+^ level (Resentini *et al*., 2021). It is clear that these new tools offer a wealth of new possibilities in plant research.

### 3. Fluorescent voltage sensors

Glass‐based microelectrodes are the gold standard for membrane transport studies as they have an excellent sensitivity, fast response times and can also be used to inject currents (Blatt, 1987; Roelfsema *et al*., 2001). Because of these properties, the glass electrodes are routinely used for membrane potential recordings, as well as voltage clamp studies with plant cells that lack plasmodesmata (Blatt, 1988; Roelfsema & Prins, 1997; Becker *et al*., 2004). Nevertheless, the use of glass electrodes has the disadvantage that the sharp‐tipped electrodes have to be carefully inserted into single cells with a micromanipulator. As a consequence, only one cell at the time can be studied and the technique is not well suited for high throughput approaches.

Because of the restrictions of glass electrodes mentioned above, alternative methods with fluorescent dyes have been developed, which are for instance routinely used to study mitochondria (Li *et al*., 2020). It was shown that the dye bis‐(1,3‐dibutylbarbituric acid)‐trimethine oxonol (DiBAC_4_) can also be used to detect plasma membrane potential changes in guard cell protoplasts (Konrad & Hedrich, 2008), intact guard cells (Cho *et al*., 2012) and various cell types in sunflower stems (Zhao *et al*., 2015). However, it is difficult to determine the absolute membrane potential values for cells in intact tissues with these dyes, or control the membrane potential (voltage clamp).

Genetically encoded voltage indicators are likely to become attractive alternatives to voltage‐sensitive dyes, as their genes can be specifically targeted to a certain cell type. Various types of voltage indicators have been developed, with a focus on their usage in brain research (Knöpfel & Song, 2019). Modern versions of these sensors display changes in fluorescence intensity of *c*. 10% during action potentials of neurons. It is likely that these new sensors will find their way in plant biology, but so far only the ArcLight sensor has been tested, which showed a high sensitivity to cytosolic pH changes (Matzke & Matzke, 2015). As stimuli such as wounding, extracellular ATP, and the growth hormone auxin trigger rapid changes in the cytosolic pH (Behera *et al*., 2018; Waadt *et al*., 2020), it will be important to develop genetically encoded voltage indicators with a very low pH‐sensitivity, for the successful application in plants.

### 4. Light‐gated ion channels

**Fig. 5:**
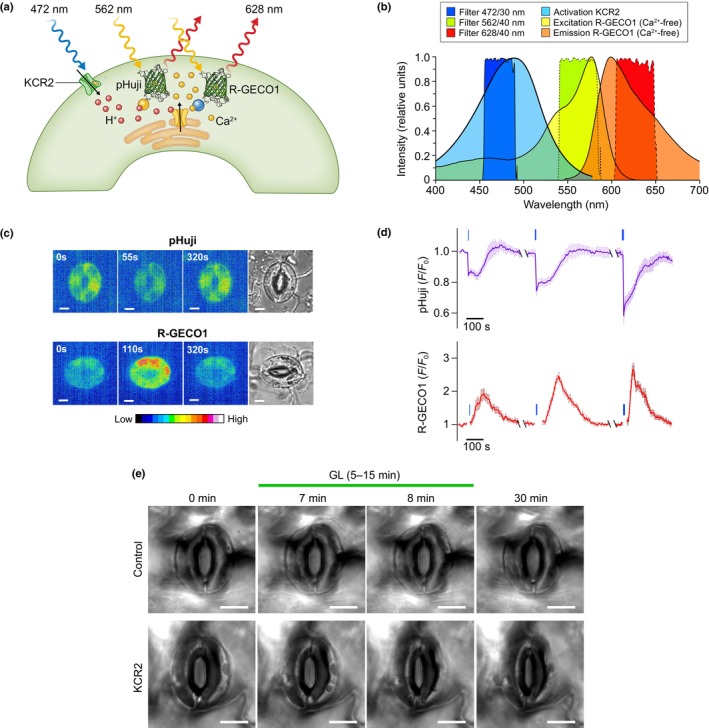
Light‐dependent initiation and detection of cytosolic H^+^‐ and Ca^2+^ responses in guard cells. (a) Schematic representation of the activation of the light‐gated KCR2 with blue light (472 nm) and the detection of cytosolic H^+^ responses with pHuji, with green excitation light (562 nm) and red emission signals (628 nm). The same filter settings were used for R‐GECO1. Note that a cytosolic H^+^ signal triggers activation of Ca^2+^ channels in the ER of guard cells (Huang *et al*., 2023). (b) Activation spectrum of KCR2 (transparent blue color) and the excitation (transparent green) and emission spectra (transparent red) of R‐GECO1, shown together with the transmission spectra of bandpass filter to stimulate KCR (472/30 nm, opaque blue color), excite pHuji and R‐GECO1 (562/40 nm, opaque yellow) and detect emission signals (628/40 nm, opaque red). (c, d) Activation of KCR with blue light pulses (1 s in (c) and 0.1, 1 and 5 s in (d)) triggers cytosolic acidification (pHuji signals), followed by a rise of the cytosolic Ca^2+^ concentration (R‐GECO1 signals). (e) Green light pulses of 0.5 s duration applied with an interval of 30 s, in the period of 5–15 min. do not affect the stomatal aperture in control leaves, but provoke closure of KCR2 expressing stomata.

Ion transport research entered a new era, with the discovery of light‐gated ion channels in *Chlamydomonas reinhardtii* (Nagel *et al*., 2002, 2003; Emiliani *et al*., 2022). In plant cells, these channels become active upon expression of a marine bacterial β‐carotene 15,15′‐dioxygenase17 (MbDio), which produces the retinal required for rhodopsin function (Zhou *et al*., 2021). These channels can be activated with light pulses and thus enable the manipulation of ion transport with a high temporal and spatial resolution (Jones *et al*., 2022; Konrad *et al*., 2023). As explained above, the *Gt*ACR1 channel provides an anion conductance that causes a depolarization in mesophyll cells (Zhou *et al*., 2021) and subsequent extrusion of anions and K^+^, as shown for guard cells (S. G. Huang *et al*., 2021). With respect to guard cells, the light‐activated KCR2 channel also can provoke K^+^ extrusion, via the H^+^‐dependent release of Ca^2+^ from the ER (Fig. 5), which triggered the activation of SLAC1 and SLAH3 (Huang *et al*., 2023).

It is likely that in addition to these channels that conduct anions (*Gt*ACR1) and H^+^ (KCR2), other rhodopsin‐type channels will become available soon and expand the possibilities to manipulate ion currents in plants. In addition to the channelrhodopsins, the light‐stimulated and voltage‐independent K^+^ channel (BLINK1) also has been expressed in guard cells (Papanatsiou *et al*., 2019). Stimulation of BLINK 1 enhanced the speed of stomatal movements, which shows that an increase in K^+^ conductance can enhance K^+^ uptake and release in guard cells. However, BLINK1 did not affect the direction of stomatal movement, which is in line with the view that anion channels determine the direction of stomatal movement (opening vs closure), as explained above (Box 2; Fig. 1).

The light‐gated channels also will offer unique possibilities to study loading of phloem tubes and xylem vessels. Just as explained for the genetically encoded ion indicators, light‐gated channels can be expressed specifically in phloem, or xylem parenchyma cells. This would enable manipulation of the membrane potential in specific cell types and study the impact on long‐distance transport of nutrients. If a blue light‐activated ion channel is chosen, it can be used in combination with red light‐emitting Ca^2+^ and pH indicators R‐GECO1 and pHuji (Fig. 5; Huang *et al*., 2023). With the use of such a combination of actors and sensors it should be possible to shed light on the long‐distance ion transport systems that have remained mysterious so far.

## Competing interests

None declared.

## Author contributions

DH, FM, CF, A‐AV and MRGR contributed to writing of the text. Figs 1, 2, 3, 5 were designed by MRGR, and Fig. 4 by DH and A‐AV. The analysis of phylogenetic relationships of ion‐channels was conducted by CF and A‐AV.

## Supporting information


**Fig. S1** Phylogenetic relationship of plant voltage‐gated cNBD K^+^ channels.
**Fig. S2** Phylogenetic relationship of ALMT channels.
**Fig. S3** Phylogenetic relationship of plant voltage‐gated Maxi‐K/BK/K_Ca_ (BK) channels.
**Fig. S4** Phylogenetic relationship of plant the Two‐Pore K^+^ TPK/KCO K^+^ channels.
**Fig. S5** Phylogenetic relationship of SLAC/SLAH channels.

**Table S1** Information on sources used to generate the phylogenetic trees.
Please note: Wiley is not responsible for the content or functionality of any Supporting Information supplied by the authors. Any queries (other than missing material) should be directed to the *New Phytologist* Central Office.
