# Supplementary material for: Potassium extrusion by plant cells: evolution from an emergency valve to a driver of long‐distance transport
Source: New Phytol. 2024 Oct 27;245(1):69–87. doi: 10.1111/nph.20207 (PMC11617655; doi:10.1111/nph.20207)
Supplement: Supplementary file 1 — Fig. S1 Phylogenetic relationship of plant voltage‐gated cNBD K+ channels. Fig. S2 Phylogenetic relationship of ALMT channels. Fig. S3 Phylogenetic relationship of plant voltage‐gated Maxi‐K/BK/KCa (BK) channels. Fig. S4 Phylogenetic relationship of plant the Two‐Pore K+ TPK/KCO K+ channels. Fig. S5 Phylogenetic relationship of SLAC/SLAH channels. [file NPH-245-69-s002.pdf]

New Phytologist **Supporting Information**

Article title: Potassium extrusion by plant cells: evolution from an emergency valve to a driver of long-distance transport

Authors: Dorsaf Hmidi, Florence Muraya, Cécile Fizames, Anne-Aliénor Véry, and M. Rob G. Roelfsema

Article acceptance date: 15 August 2024

The following Supporting Information is available for this article:

Fig. S1 Phylogenetic relationship of plant voltage-gated cNBD K^+ channels.

Fig. S2 Phylogenetic relationship of ALMT channels

Fig. S3 Phylogenetic relationship of plant voltage-gated Maxi-K/BK/K_{Ca} (BK) channels.

Fig. S4 Phylogenetic relationship of plant Two-Pore K^+ TPK/KCO K^+ channels.

Fig. S5 Phylogenetic relationship of SLAC/SLAH channels.

Table S1 Information on sources used to generate the phylogenetic trees.

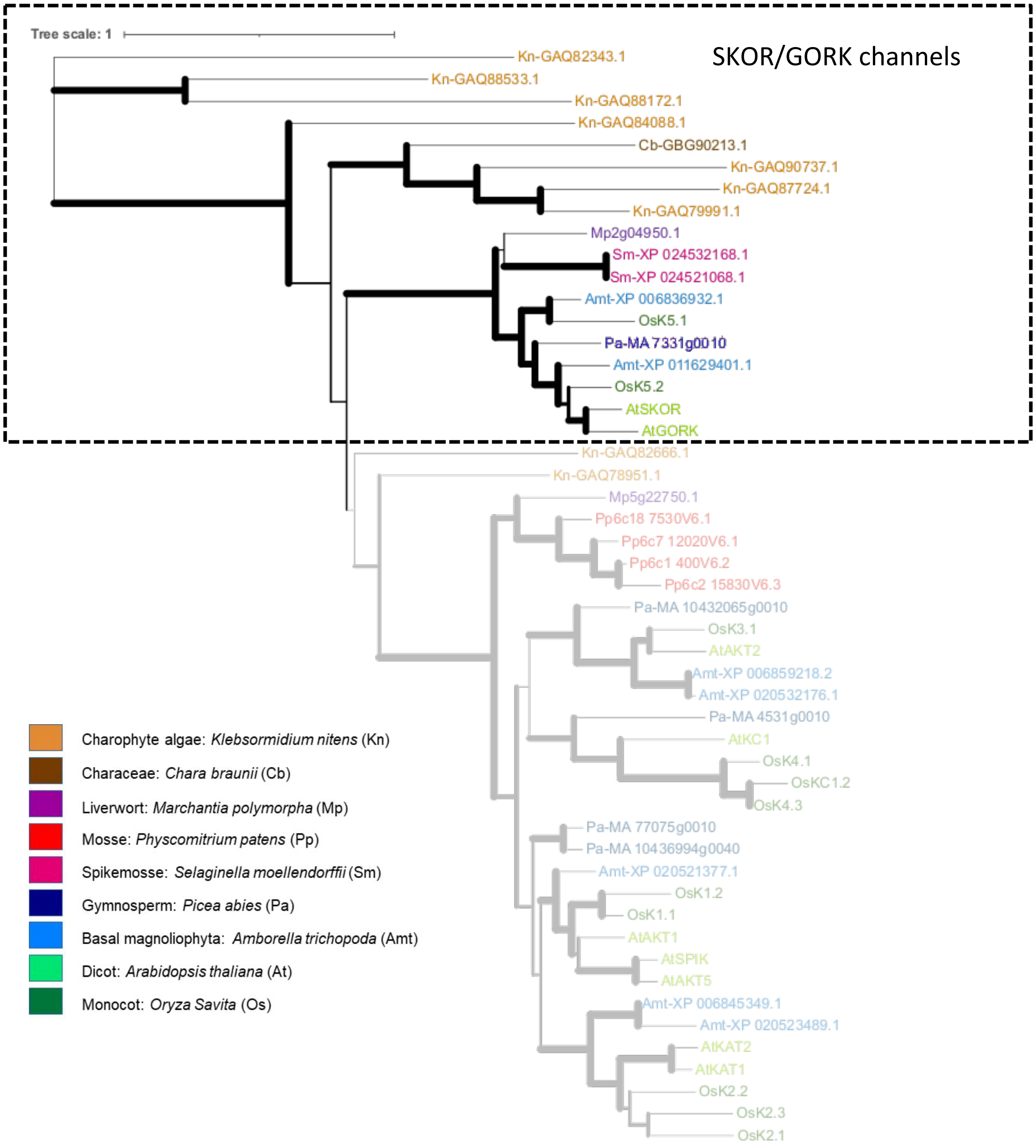

Figure S1 Phylogenetic relationship of plant voltage-gated cNBD K^+ channels. Maximum likelihood trees were built with the amino acid sequences of the voltage-gated cNBD (cyclic-nucleotide-binding domain)-containing K^+ channels (Jegla *et al.*, 2018), previously named "plant Shakers" (Véry *et al.*, 2014). The sequences were gathered from the 9 species shown in the legend. The thickness of the tree branches reflects the bootstrap value. For tree building, a multiple alignment of the sequences was created using MAFFT and gaps and misaligned segments were removed using Gblocks in Seaview (Galtier *et al.*, 1996) prior to phylogenetic analysis. Maximum likelihood analysis was performed using PHYML (Guindon & Gascuel, 2003) and bootstrap values were determined from 100 replicates.

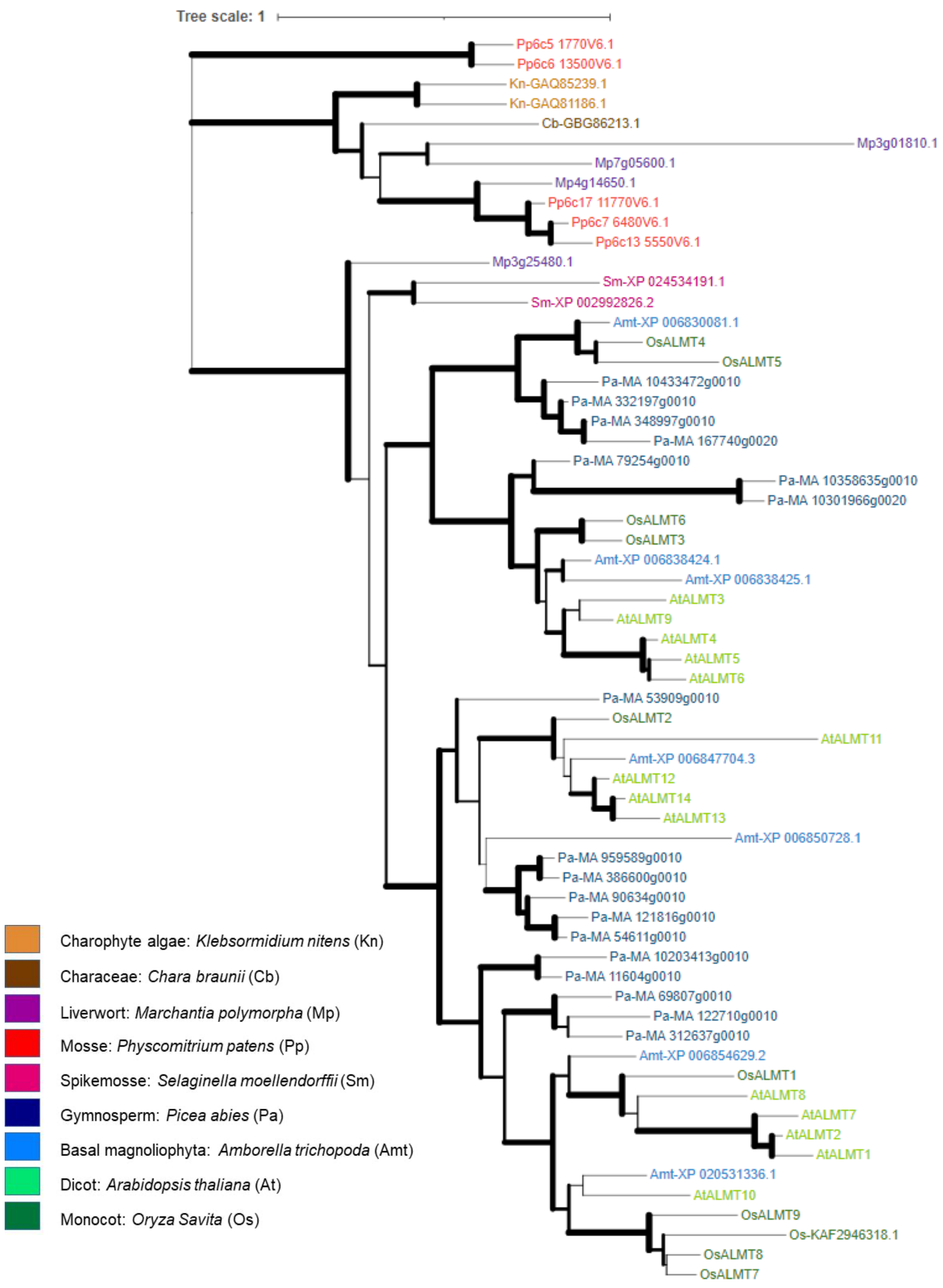

Figure S2 Phylogenetic relationship of ALMT channels (Dreyer *et al.*, 2012; Sharma *et al.*, 2016). Maximum likelihood trees were built with the amino acid sequences of ALMT channels, from the 9 species shown in the legend. The thickness of the tree branches reflects the bootstrap value. For tree building, a multiple alignment of the sequences was created using MAFFT and gaps and misaligned segments were removed using Gblocks in Seaview (Galtier *et al.*, 1996) prior to phylogenetic analysis. Maximum likelihood analysis was performed using PHYML (Guindon & Gascuel, 2003) and bootstrap values were determined from 100 replicates.

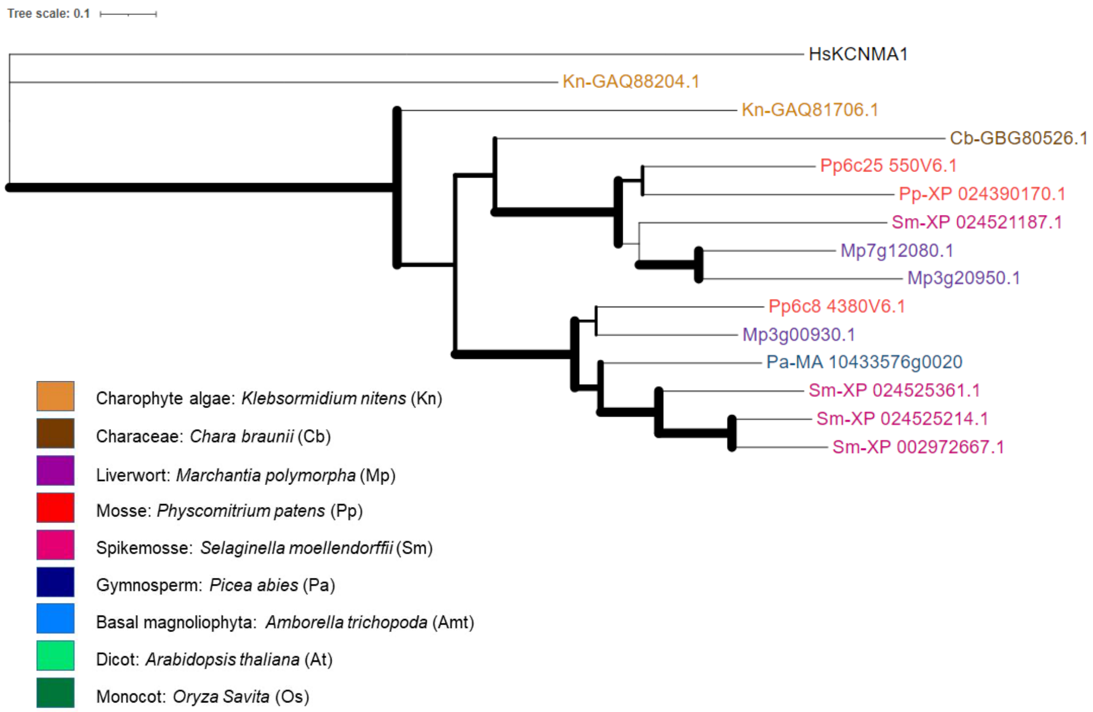

Figure S3 Phylogenetic relationship of plant voltage-gated Maxi-K/BK/K_{Ca} (BK) channels. Maximum likelihood trees were built with the amino acid sequences of plant BK channels and the human BK channel, *Hs*KCNMA1 (Sancho & Kyle, 2021). The sequences were gathered from the 9 species shown in the legend. The thickness of the tree branches reflects the bootstrap value. For tree building, a multiple alignment of the sequences was created using MAFFT and gaps and misaligned segments were removed using Gblocks in Seaview (Galtier *et al.*, 1996) prior to phylogenetic analysis. Maximum likelihood analysis was performed using PHYML (Guindon & Gascuel, 2003) and bootstrap values were determined from 100 replicates.

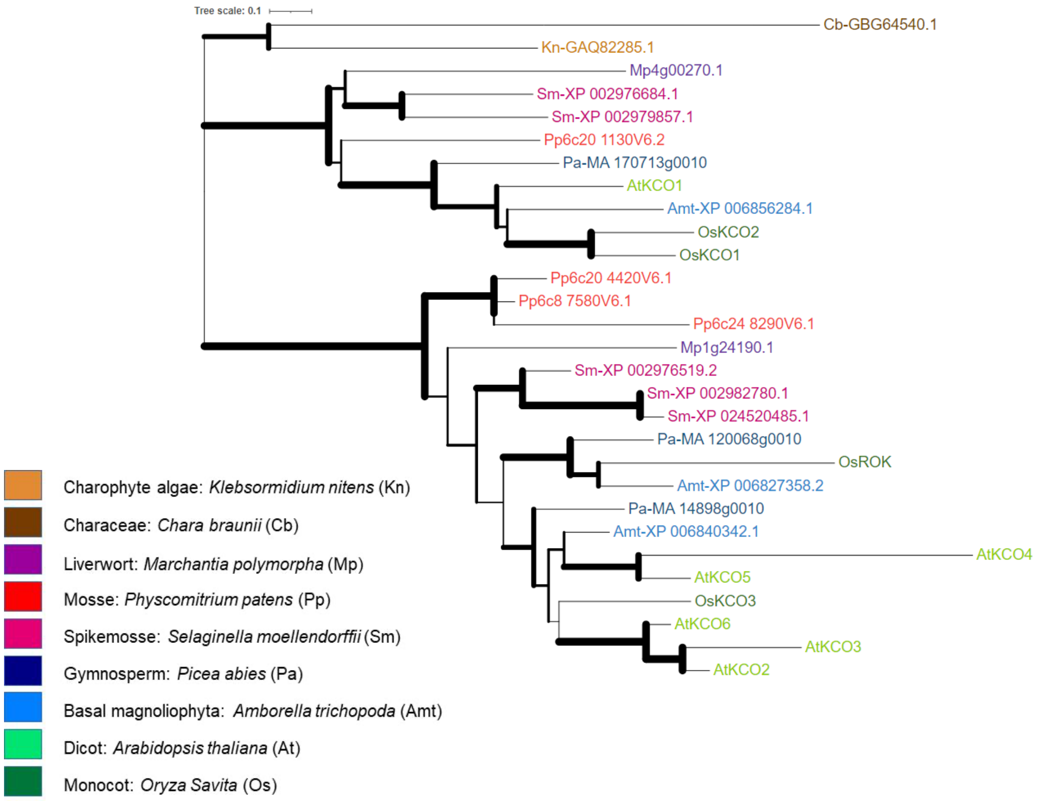

Figure S4 Phylogenetic relationship of plant Two-Pore K^+ TPK/KCO K^+ channels (Maathuis, 2011; Dabravolski & Isayenkov, 2021). Maximum likelihood trees were built with the amino acid sequences of plant TPK/KCO channels, from the 9 species shown in the legend. The thickness of the tree branches reflects the bootstrap value. For tree building, a multiple alignment of the sequences was created using MAFFT and gaps and misaligned segments were removed using Gblocks in Seaview (Galtier *et al.*, 1996) prior to phylogenetic analysis. Maximum likelihood analysis was performed using PHYML (Guindon & Gascuel, 2003) and bootstrap values were determined from 100 replicates.

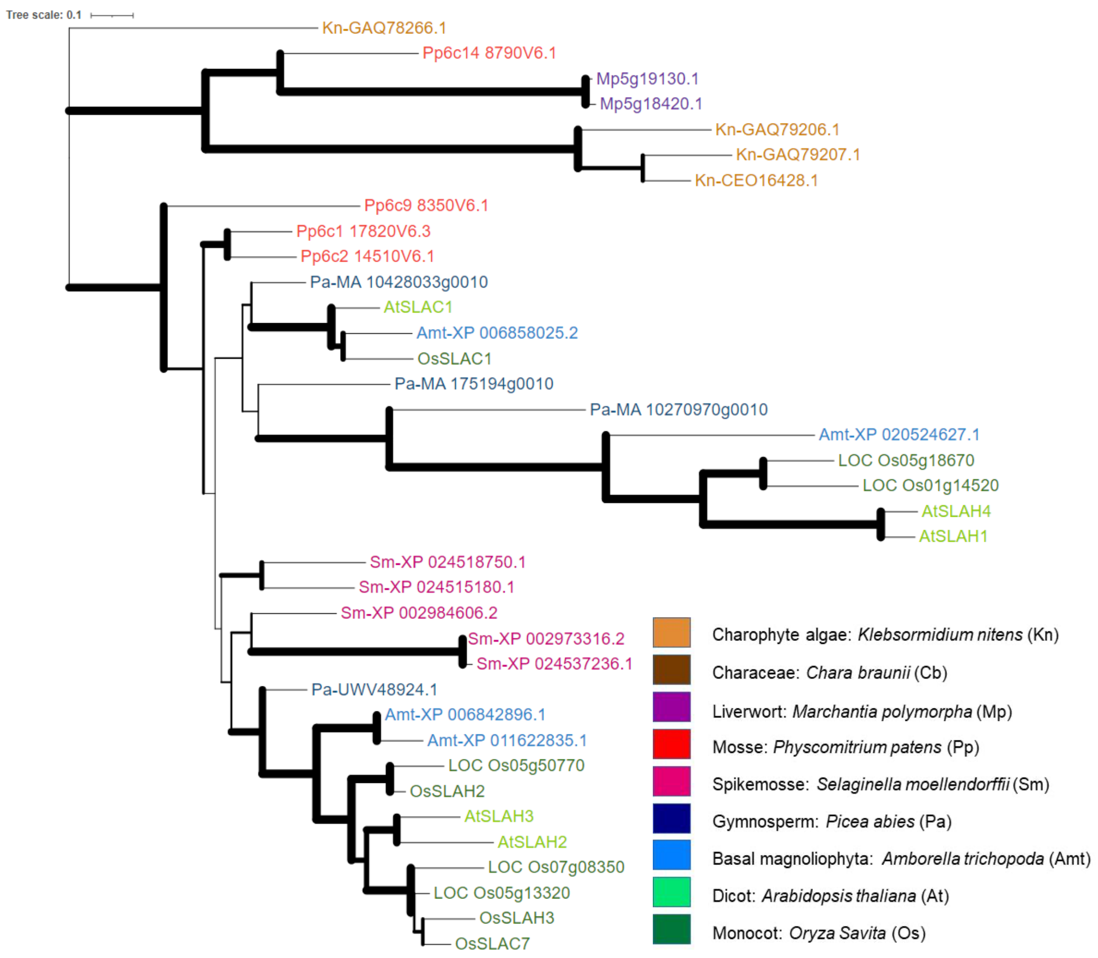

Figure S5 Phylogenetic relationship of SLAC/SLAH channels (Dreyer *et al.*, 2012). Maximum likelihood trees were built with the amino acid sequences of SLAC/SLAH channels, from the 9 species shown in the legend. The thickness of the tree branches reflects the bootstrap value. For tree building, a multiple alignment of the sequences was created using MAFFT and gaps and misaligned segments were removed using Gblocks in Seaview (Galtier *et al.*, 1996) prior to phylogenetic analysis. Maximum likelihood analysis was performed using PHYML (Guindon & Gascuel, 2003) and bootstrap values were determined from 100 replicates.

References

Dabravolski SA, Isayenkov SV. 2021. New Insights into plant TPK ion channel evolution. *Plants-Basel* **10**(11): 15.

Dreyer I, Gomez-Porras JL, Riaño-Pachón DM, Hedrich R, Geiger D. 2012. Molecular evolution of slow and quick anion channels (SLACs and QUACs/ALMTs). *Frontiers in Plant Science* **3**: 12.

Galtier N, Gouy M, Gautier C. 1996. SEAVIEW and PHYLO_WIN: Two graphic tools for sequence alignment and molecular phylogeny. *Computer Applications in the Biosciences* **12**(6): 543-548.

Guindon S, Gascuel O. 2003. A simple, fast, and accurate algorithm to estimate large phylogenies by maximum likelihood. *Systematic Biology* **52**(5): 696-704.

Jegla T, Busey G, Assmann SM. 2018. Evolution and structural characteristics of plant voltage-gated K^+ channels. *Plant Cell* **30**(12): 2898-2909.

Maathuis FJM. 2011. Vacuolar two-pore K+ channels act as vacuolar osmosensors. *New Phytologist* **191**(1): 84-91.

Sancho M, Kyle BD. 2021. The large-conductance, calcium-activated potassium channel: A big key regulator of cell physiology. *Frontiers in Physiology* **12**: 16.

Sharma T, Dreyer I, Kochian L, Piñeros MA. 2016. The ALMT family of organic acid transporters in plants and their involvement in detoxification and nutrient security. *Frontiers in Plant Science* **7**: 12.

Véry AA, Nieves-Cordones M, Daly M, Khan I, Fizames C, Sentenac H. 2014. Molecular biology of K^+ transport across the plant cell membrane: What do we learn from comparison between plant species? *Journal of Plant Physiology* **171**(9): 748-769.
